# Supplementary material for: Focal Cryo-Immunotherapy with Intratumoral IL-12 Prevents Recurrence of Large Murine Tumors
Source: Cancers (Basel). 2023 Apr 8;15(8):2210. doi: 10.3390/cancers15082210 (PMC10137033; doi:10.3390/cancers15082210)
Supplement: Supplementary file 1 [file cancers-15-02210-s001.zip › cancers-2213781-supplementary.pdf]

Supplementary Materials

All media protocols are for 500 mL total volume:

Table S1. Basic Media (MB49, LLC1, Panc02)
Remove 60 mL of DMEM 4.5 g/L glucose & sodium pyruvate, without L-glutamine (Corning #15-013-CV)

	Stock concentration	Final concentration	Volume to add (mL)
Heat-inactivated FBS (HI FBS)	100%	10%	50
L-glutamine	200 mM	2 mM	5
Penicillin/streptomycin	10,000 U/mL	100 U/mL	5

Table S2. MC38 Media
Remove 75 mL of DMEM 4.5 g/L glucose & sodium pyruvate, without L-glutamine (Corning #15-013-CV)

	Stock concentration	Final concentration	Volume to add (mL)
Heat-inactivated FBS (HI FBS)	100%	10%	50
Non-essential amino acids (NEAA)	100X	1X	5
Sodium pyruvate	100 mM	1 mM	5
L-glutamine	200 mM	2 mM	5
HEPES buffer	1 M	10 mM	5
Penicillin/streptomycin	10,000 U/mL	100 U/mL	5

Table S3. B16.F10 Media
Remove 75 mL of DMEM 4.5 g/L glucose & sodium pyruvate, without L-glutamine (Corning #15-013-CV)

	Stock concentration	Final concentration	Volume to add (mL)
Heat-inactivated FBS (HI FBS)	100%	10%	50
Sodium bicarbonate	7.5 w/v%	0.15 w/v%	10
L-glutamine	200 mM	4 mM	10
Sodium pyruvate	100 mM	1 mM	5

Table S4. T Cell Media
Remove 75 mL of RPMI 1640 without L-glutamine (Corning #15-040-CV)

	Stock concentration	Final concentration	Volume to add (mL)
Heat-inactivated FBS (HI FBS)	100%	10%	50
Non-essential amino acids (NEAA)	100X	1X	5
Sodium pyruvate	100 mM	1 mM	5
L-glutamine	200 mM	2 mM	5
HEPES buffer	1 M	10 mM	5
Penicillin/streptomycin	10,000 U/mL	100 U/mL	5
2-mercaptoethanol	14.3 M	55 uM	1.92 uL

Supplementary Figures

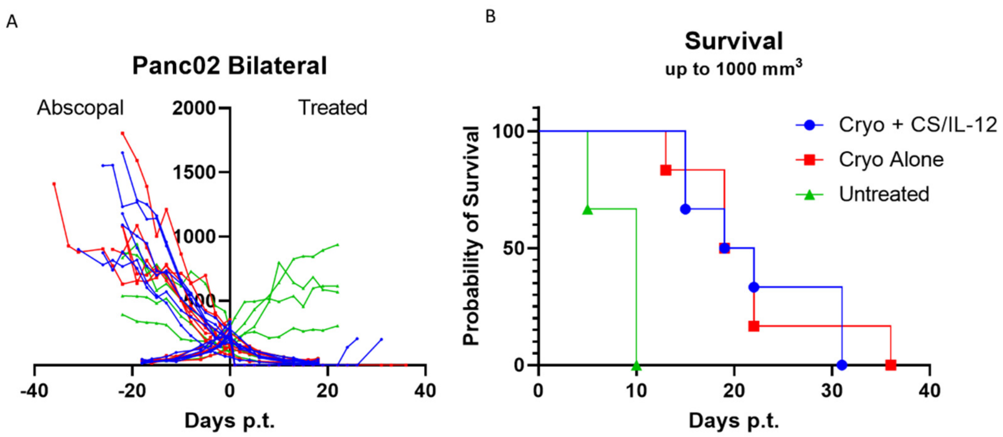

Figure S1. Panc02 cells were implanted subcutaneously on the right ($1x10^6$ cells) and left ($0.5x10^6$ cells) flanks and the larger of the two tumors was treated with either CA alone or CA + CS/IL-12 when it measured between 200 – 300 mm^3. Tumor volume (A) and survival (B) were monitored. The threshold to euthanize subjects was a total tumor burden of 1000 mm^3.

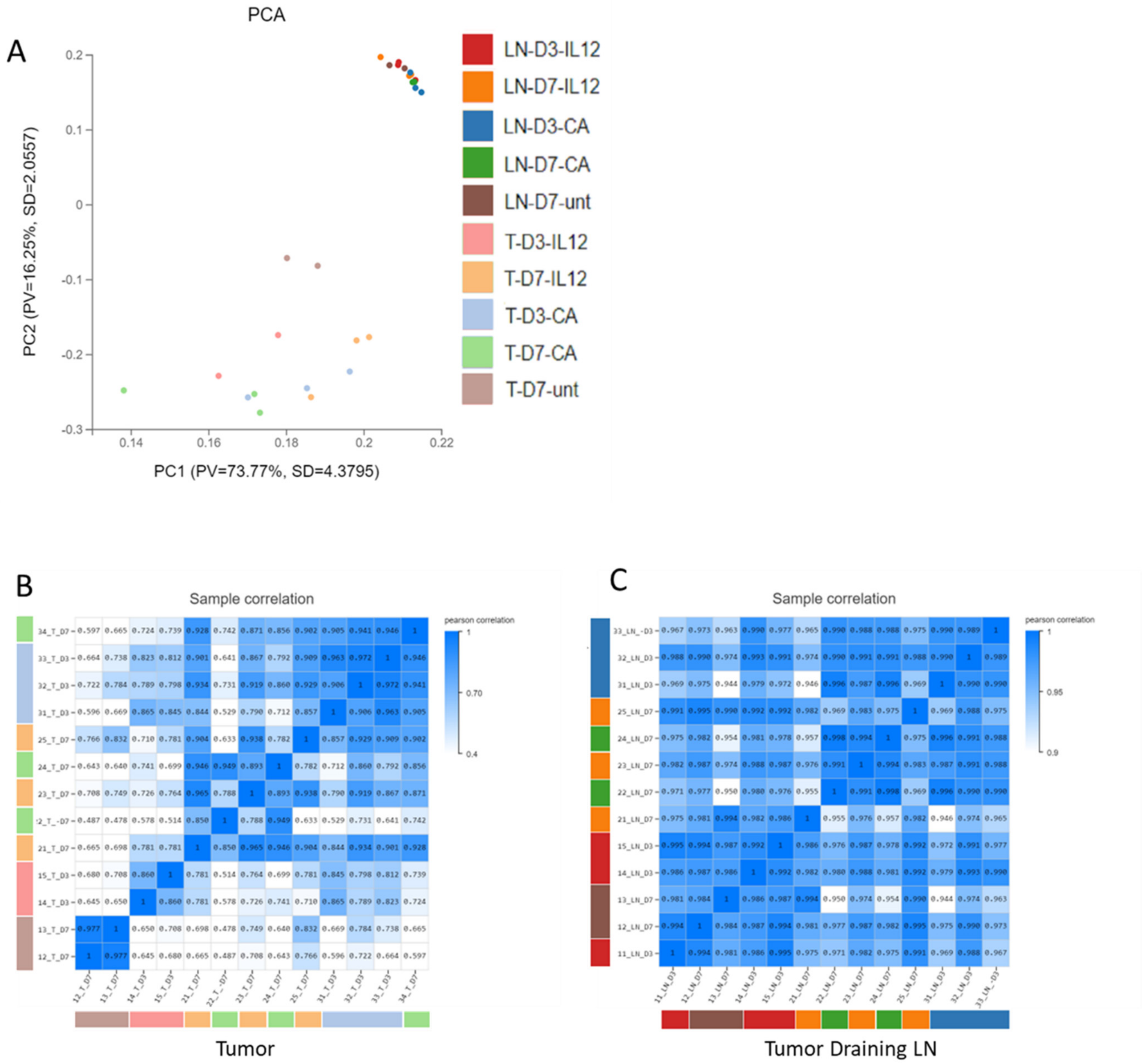

Figure S2. (A) Primary component analysis was performed on all samples across the transcriptome with a predictive value of 73.77% for the first component. Sample correlation heatmaps for all (B) tumor and (C) LN samples using Pearson's correlation and TPM as the unit of measure where all genes with an expression of NA were excluded. Legend: Dark colors indicate tumor draining LNs samples, light colors indicate tumor samples. Warm colors (red and orange) represent samples from the CA + CS/IL-12 group, cool colors (blue and green) represent samples from the CA only group. Day 3 (D3) samples are denoted in primary colors (read and blue) and day 7 (D7) samples denoted in secondary colors (orange and green). Brown represents samples from the untreated group. Abbreviations: TPM, transcripts per kilobase million; T, tumor; LN, tumor draining lymph node; D3, day 3 after treatment; D7, day 7 after treatment; CA, treatment with cryoablation alone; IL12, treatment with cryoablation plus intratumoral interleukin-12 formulated in chitosan (CA + CS/IL-12).

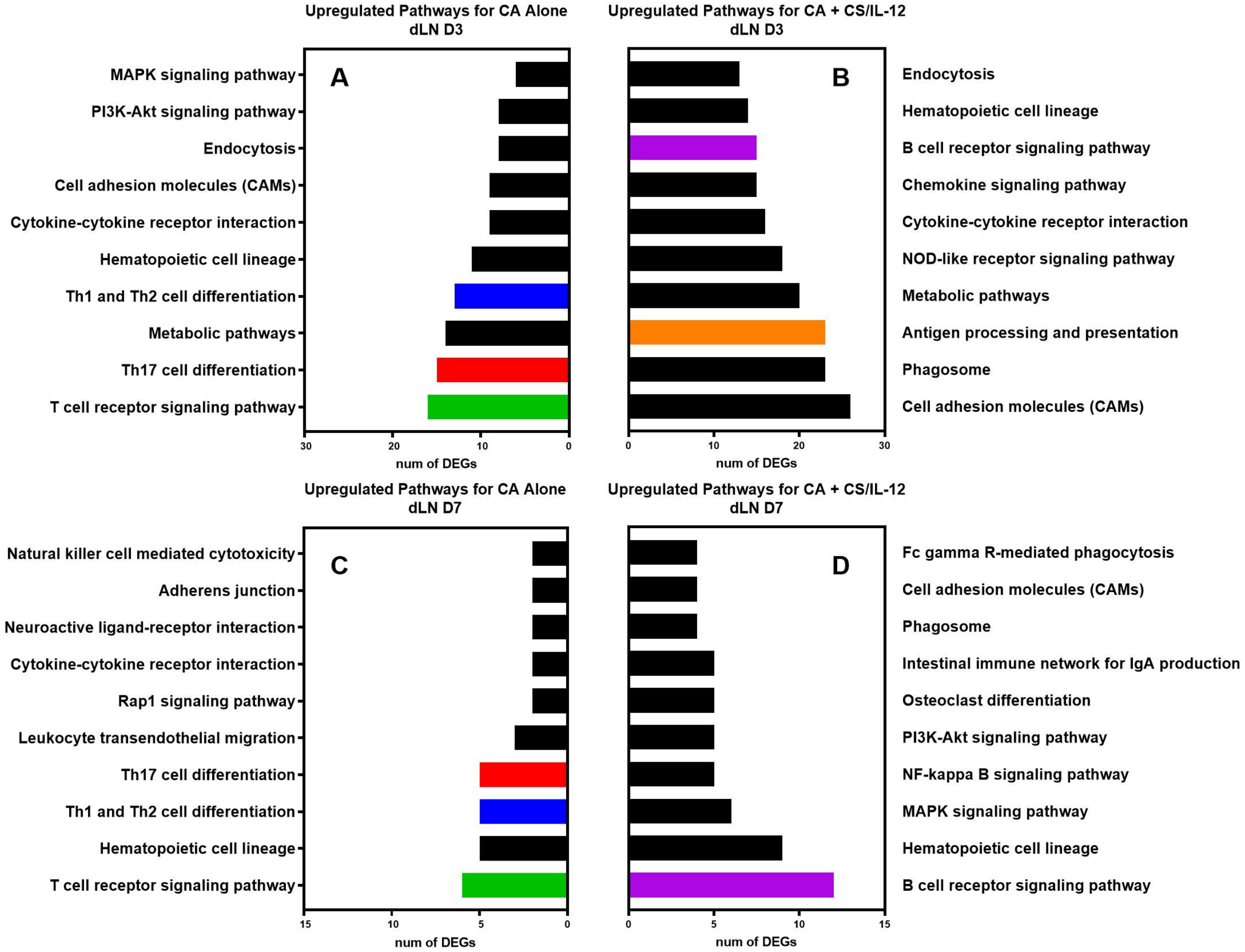

Figure S3. Gene annotation was performed for the genes upregulated in each treatment group based on the heatmaps from Fig 6B-C. For day 3 post treatment, the top 10 KEGG pathways for CA Alone (A) and CA + CS/IL-12 (B) had some overlap, for example, with the hematopoietic cell lineage, cell adhesion molecules (CAMs), and endocytosis. For day 7 post treatment, the top pathways for CA Alone (C) were T cell receptor signaling, hematopoietic cell lineage, and Th1 and Th2 cell differentiation. For CA + CS/IL-12 on day 7 (D), the top pathways were B cell receptor signaling, hematopoietic cell lineage, and MAPK signaling. The solid colored bars correspond to the pathways of interest from Fig 6F-G.

Tumor D7

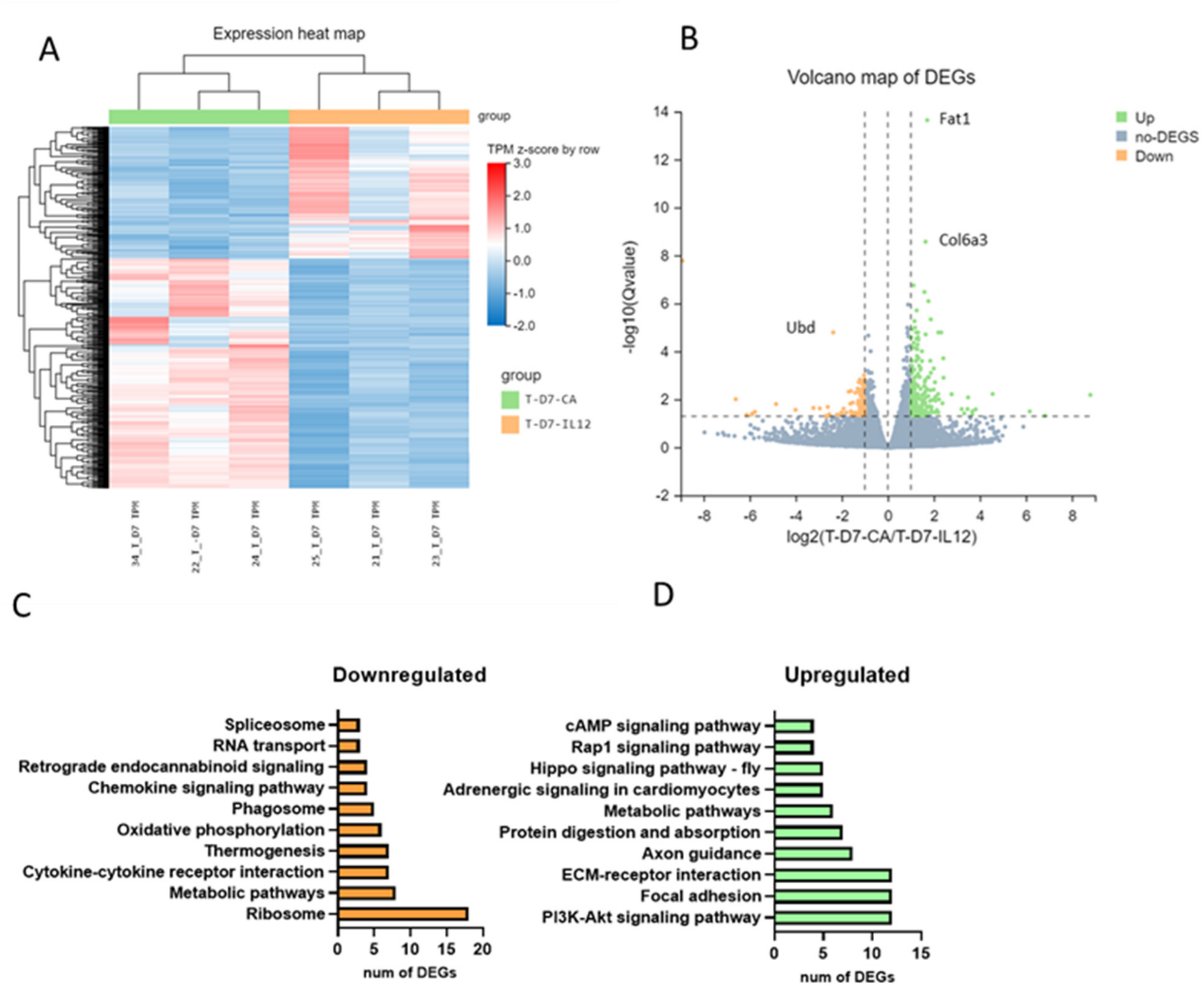

Figure S4. (A) Heatmap analysis of D7 tumor samples reveals significant differences in gene expression between the treatment groups. (B) A volcano plot of the differentially expressed genes reveals Fat1 and Col6a3 to be significantly upregulated in the CA alone group while Ubd is expressed more highly in the CA + CS/IL-12 treated group. Further classification analysis of KEGG pathways was performed separately for significantly downregulated genes (C) and upregulated genes (D) from panel B, with the top 10 pathways shown. Abbreviations: T, tumor; LN, tumor draining lymph node; D7, day 7 after treatment; CA, treatment with cryoablation alone; IL12, treatment with cryoablation plus intratumoral interleukin-12 formulated in chitosan (CA + CS/IL-12)

Draining Lymph Node and Tumor D7

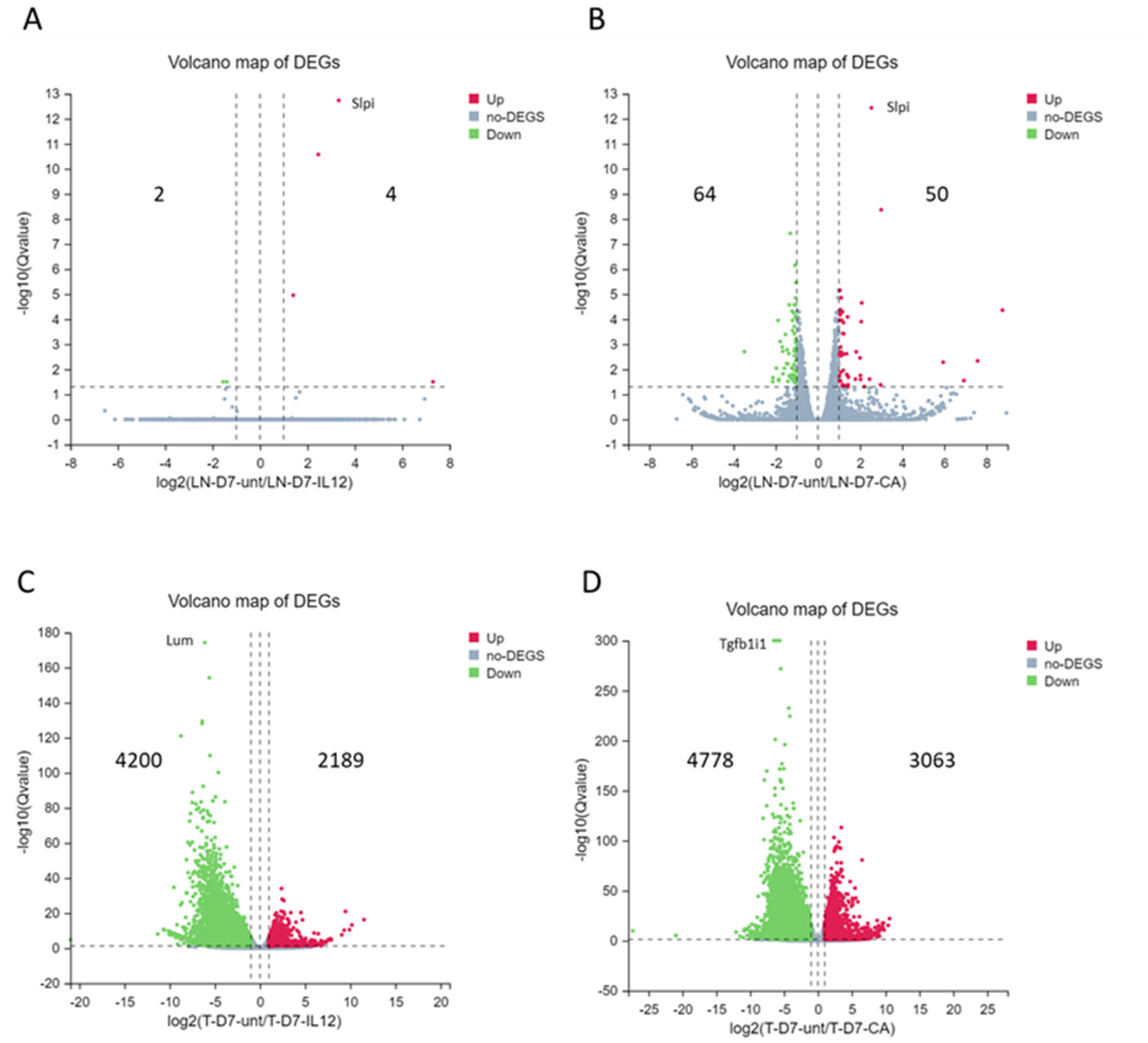

Figure S5. Differential gene expression analysis for treatment groups compared to untreated controls shown as volcano plots was performed on (A,B) LN samples and (C,D) tumor samples. The threshold for significant difference was |log2FC| >= 1 and Qvalue <= 0.05. Abbreviations: T, tumor; LN, tumor draining lymph node; D7, day 7 after treatment; CA, treatment with cryoablation alone; IL12, treatment with cryoablation plus intratumoral interleukin-12 formulated in chitosan; unt, untreated tumor; Slpi, secretory leukocyte peptidase inhibitor; Lum, lumican; Tgfb1i1, transforming growth factor beta 1 induced transcript 1.

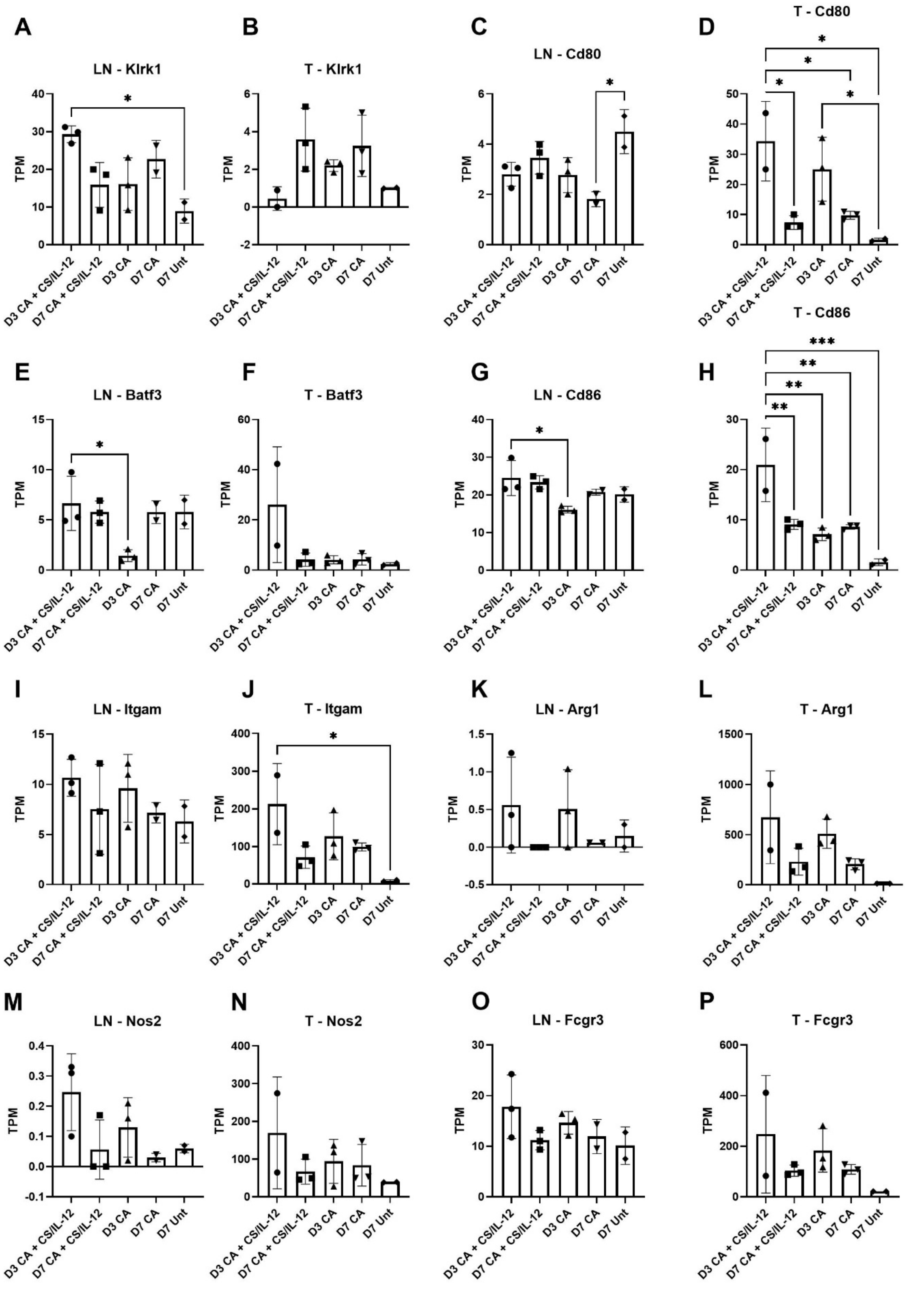

Figure S6. Dendritic cell, NK cell, and macrophage associated gene expression in the dLN and tumor. Klrk1 (NKG2D) is a mediator of NK-associated cytotoxicity (A, B). CD80 (C, D) and CD86 (G, H) are costimulatory receptors found on DCs and macrophages. BATF3 is a marker for cross presenting DCs (E, F). Itgam (CD11b) is a general macrophage marker (I, J), while Arg1 (Arginase) (K, L) and Nos2 (iNOS) (M, N) are markers for suppressive M2-like macrophages. Fcgr3 (CD16) is a marker for neutrophils (O, P). Statistical significance was calculated using a one-way analysis of variance with Tukey multiple comparison correction. ns - not significant; *p<0.05; **p<0.01; ***p<0.001; ****p<0.0001.

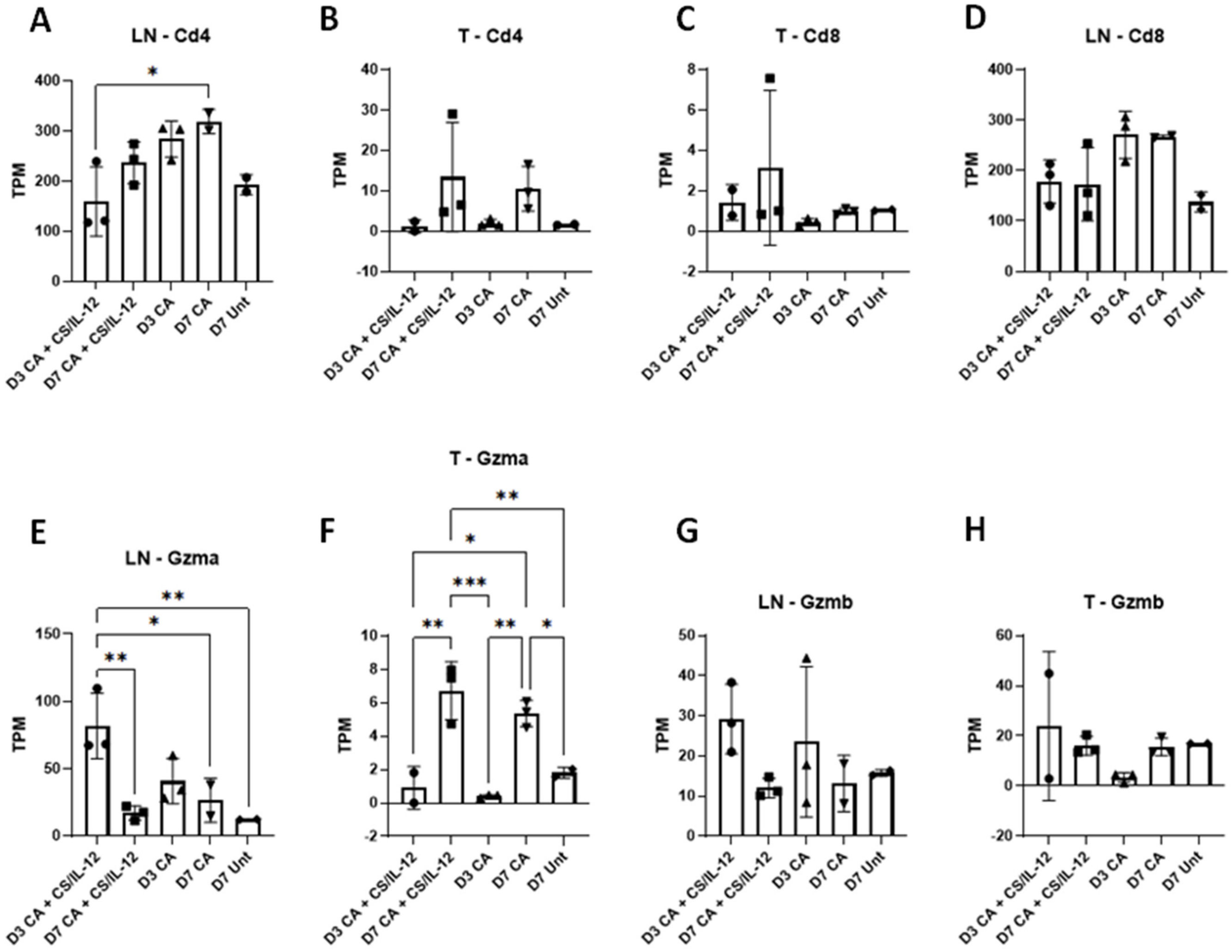

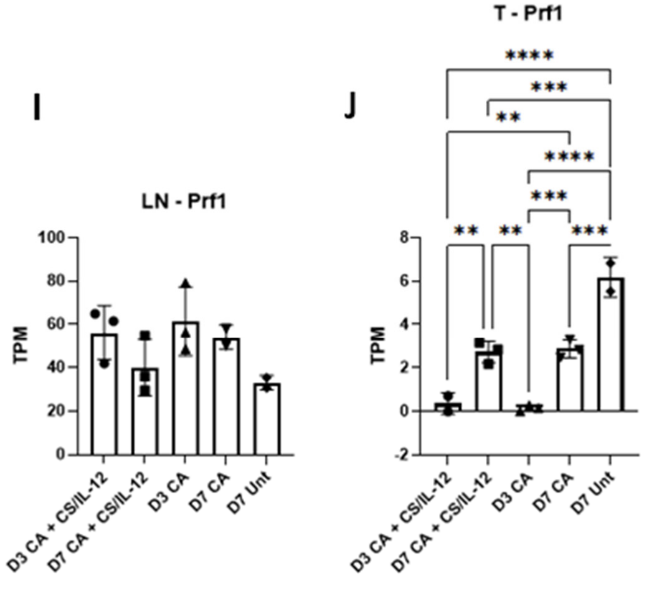

Figure S7. T cell associated gene expression in the dLN and tumor. Cd4 (A, B) and Cd8 (C, D) are markers for helper and cytotoxic T cells, respectively. Gzma (E, F), Gzmb (G, H), and Prf1 (Perforin) (I, J) are effectors of cytotoxic function. Statistical significance was calculated using a one-way analysis of variance with Tukey multiple comparison correction. ns - not significant; *p<0.05; **p<0.01; ***p<0.001; ****p<0.0001.
